# Investigation of New Orexin 2 Receptor Modulators Using *In Silico* and In Vitro Methods

**DOI:** 10.3390/molecules23112926

**Published:** 2018-11-09

**Authors:** Jana Janockova, Rafael Dolezal, Eugenie Nepovimova, Tereza Kobrlova, Marketa Benkova, Kamil Kuca, Jan Konecny, Eva Mezeiova, Michaela Melikova, Vendula Hepnarova, Avi Ring, Ondrej Soukup, Jan Korabecny

**Affiliations:** 1 Biomedical Research Centre, University Hospital Hradec Kralove, Sokolska 581, 500 05 Hradec Kralove, Czech Republic; jana.janockova@fnhk.cz (J.J.); Rafael.dolezal@fnhk.cz (R.D.); tereza.kobrlova@fnhk.cz (T.K.); marketa.benkova@fnhk.cz (M.B.); kamil.kuca@fnhk.cz (K.K.); jan.konecny@fnhk.cz (J.K.); eva.mezeiova@fnhk.cz (E.M.); vendula.hepnarova@unob.cz (V.H.); 2 Department of Chemistry, Faculty of Science, University of Hradec Kralove, Rokitanskeho 62, 500 03 Hradec Kralove, Czech Republic; eugenie.nepovimova@uhk.cz (E.N.); michaela.melikova@upol.cz (M.M.); 3 Center for Basic and Applied Research, University of Hradec Kralove, Rokitanskeho 62, 500 03 Hradec Kralove, Czech Republic; 4 Department of Toxicology and Military Pharmacy, Faculty of Military Health Sciences, University of Defence, Trebesska 1575, 500 05 Hradec Kralove, Czech Republic; 5 Norwegian Defence Research Establishment, Gunnar Randersvei 42, 2007 Kjeller, Norway; avi.ring@ffi.no

**Keywords:** orexin A, suvorexant, orexin receptor modulators, narcolepsy, structure-based virtual screening

## Abstract

The neuropeptides, orexin A and orexin B (also known as hypocretins), are produced in hypothalamic neurons and belong to ligands for orphan G protein-coupled receptors. Generally, the primary role of orexins is to act as excitatory neurotransmitters and regulate the sleep process. Lack of orexins may lead to sleep disorder narcolepsy in mice, dogs, and humans. Narcolepsy is a neurological disorder of alertness characterized by a decrease of ability to manage sleep-wake cycles, excessive daytime sleepiness, and other symptoms, such as cataplexy, vivid hallucinations, and paralysis. Thus, the discovery of orexin receptors, modulators, and their causal implication in narcolepsy is the most important advance in sleep-research. The presented work is focused on the evaluation of compounds **L1**–**L11** selected by structure-based virtual screening for their ability to modulate orexin receptor type 2 (OX2R) in comparison with standard agonist orexin-A together with their blood-brain barrier permeability and cytotoxicity. We can conclude that the studied compounds possess an affinity towards the OX2R. However, the compounds do not have intrinsic activity and act as the antagonists of this receptor. It was shown that **L4** was the most potent antagonistic ligand to orexin A and displayed an IC_50_ of 2.2 µM, offering some promise mainly for the treatment of insomnia.

## 1. Introduction

The dorsolateral hypothalamic neuromodulators, orexin A and orexin B, are also known as hypocretin 1 and hypocretin 2, respectively [1]. Orexin A consists of 33 amino acids with two disulphide bridges, while orexin B is a linear 28-residue neuropeptide [2,3]. Orexins regulate various physiological functions, such as the sleep-wake cycles, food intake, pleasure-seeking behaviour etc. [4]. The double designation of these peptides reflects their discovery by two independent groups. After nucleotide sequencing, de Lecea et al. named these peptides as “hypocretins” due to their similarity to the intestinal hormone, secretin, and to the localization of the neurons producing such peptides in the hypothalamus [2]. On the other hand, Sakurai et al., isolated and named these neuromodulators as “orexins” because of their evident orexigenic (appetite-stimulating) activity, which was revealed by methods exploiting receptor cloning [3]. Orexins elicit their response by binding to particular receptors—orexin receptor 1 (OX1R) and orexin receptor 2 (OX2R) [5]. Both receptors belong among the G protein-coupled receptors [6]. Recently, the binding affinity of orexin peptides to OX1R and OX2R has been determined. The results affirmed that orexin-A binds both OX1R and OX2R with the same affinity, whereas orexin-B displays higher selectivity for OX2R [7]. 

Recent discoveries in neurology have indicated that the pathophysiology of human narcolepsy is strongly associated with the loss of lateral hypothalamic neurons producing orexins [8]. According to several studies, orexigenic neurons are a target of immune system cells, prompting an autoimmune aetiology of the disease [9]. Narcolepsy manifests by chronic sleepiness and at least one additional symptom, such as cataplexy (sudden episodes of partial or complete paralysis of voluntary muscles), vivid hallucinations, or total paralysis [10]. The occurrence of this disorder is quite low (one in 2000 people) and there is no causal treatment for such patients [10]. They are referred to symptomatic therapy involving modafinil, sodium oxybate, and/or antidepressants to treat cataplexy [11]. Modafinil acts in the central nervous system (CNS) as a dopamine reuptake inhibitor, which is indirectly coupled with the production of orexins and histamine [12]. The mechanism of oxybate action remains unrevealed, although it is supposed that oxybate could modulate gamma-aminobutyric acid receptors B [13]. Since the aforementioned therapeutics represent a group of stimulants with an increased risk of addiction, specifically designed orexin receptor agonists are more likely to become the ideal therapeutic option for orexin-deficient narcoleptics [1,14,15,16,17]. However, such compounds are still not available for humans [1]. It is also worth mentioning that viruses invading the brain along the olfactory route could target OXRs and cause narcolepsy syndrome [18]. From this point of view, another relevance for the development of OXR agonists to alleviate syndromes associated with the virus disorder exists [19].

Thus, loss of orexinergic neurons is associated with severe sleepiness and inability to maintain wakefulness. On the other hand, a role has emerged for OX1R/OX2R receptor antagonists as sleep promoters in the treatment of insomnia [20]. Accordingly, suvorexant, a non-selective OXR antagonist, has been approved to facilitate sleep induction and maintenance [21].

Accordingly, OX1R/OX2R ligands (both agonists and antagonists) are interesting targets in the drug discovery field [22]. In this work, we used a structure-based virtual screening method to find novel candidates for OX2R modulators. The 11 top-ranked ligands were purchased and tested in vitro to evaluate the results of our *in silico* approach. The proposed ligands were also assessed for their safety profile (i.e., cytotoxicity) and potential capability to cross the blood-brain barrier, as non-specific properties relevant for CNS-targeted drugs. 

## 2. Results and Discussion

The objective of the present study was to use structure-based virtual screening (SBVS) to find small lead-like molecules capable of modulating the activity of OX2R. Since both agonists and antagonists of OX2R are supposed to exhibit a significant binding affinity towards the receptor, the research focused on the discovery of such compounds that fit to the active site of the OX2R model and are fixed in this position by a strong binding energy. Based on the stepwise selection of OX2R modulators from 1,000,448 compounds by iDock and AutoDock Vina software, 11 compounds with the lowest binding energy estimates (i.e., the strongest binding affinity) were chosen for purchasing and in vitro screening of their activity in OX2R. Their chemical structures and log*P* values are depicted in Table 1. In addition, suvorexant as a known OX2R antagonist was analyzed by the same computational methodology as the compounds involved in the SBVS. The resulting binding energy estimates obtained in iDock and AutoDock Vina software are summarized in Table 2. 

From Table 2, it is obvious that nearly all simulated compounds exhibit a lower *in silico* binding energy in the OX2R model than suvorexant. Since the final molecular docking involved also torsional flexibility of amino acid residues in the active site of OX2R, the binding energy estimates given by AutoDock Vina are lower than those obtained by iDock. The lowest binding energy estimates in AutoDock Vina were observed in the compounds, **L5** (−15.4 kcal/mol), **L4** (−15.0 kcal/mol), and **L2** (−14.8 kcal/mol). 

**Figure 1:**
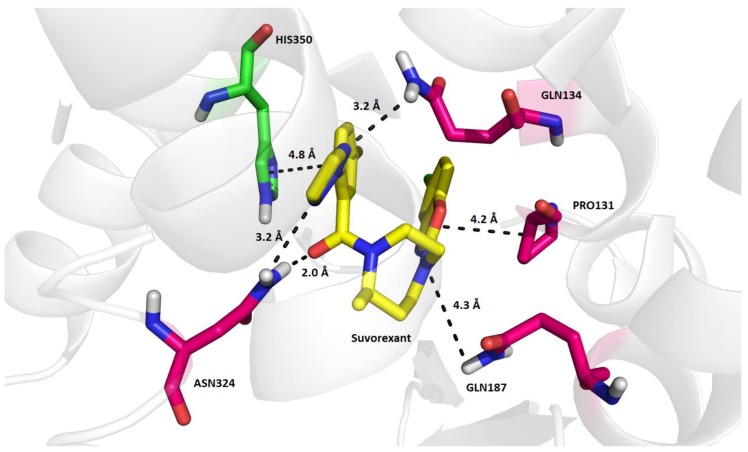
Binding mode of suvorexant in OX2R as determined by X-ray in the 4S0V model. The four residues colored in magenta (Pro131, Gln134, Gln187, Asn324) are mainly responsible for binding the antagonist in the active site of OX2R. On the contrary, His350 (in green) is a member of the proposed activation OX2R tetrad (e.g., Thr111, Asp115, His350, and Tyr354) responsible for eliciting the agonistic effect [23].

Computational analyses of the X-ray model of OX2R co-crystallized with suvorexant (PDB ID: 4S0V, Figure 1) revealed that the antagonist is bound to the active site of the receptor mainly by van der Waals interactions and hydrogen bonds, intermediated by water molecules in some cases. The triazole moiety of suvorexant is stabilized in the binding mode by weak interactions with the terminal amino groups of Asn324 (3.2 Å) and Gln134 (2.0 Å). Similarly, the 1,4-diazepane moiety of suvorexant interacts by a weak hydrogen bond with the amino group of Gln187 (4.3 Å). In addition, suvorexant is fixed in the binding mode in OX2R by a weak hydrophobic interaction of its benzo[*d*]oxazole moiety with Pro131 (4.2 Å) and by a weak π-stacking of the distal benzene ring with His350 (4.8 Å). Interaction of His350 with suvorexant in 4S0V is further strengthened by a hydrogen bond intermediated by a water molecule [22]. The nearly identical binding mode of suvorexant in OX2R as that in the 4S0V model (Figure 1) was predicted by flexible molecular docking in AutoDock Vina with a binding energy estimate of −12.5 kcal/mol (comparison of the original and docked position of suvorexant resulted in a root-mean square distance (RMSD) of 0.185 Å, Figure S1). 

Importantly, interactions of OX2R modulators with the residues, Thr111, Asp115, His350, and Tyr354 (which can be denoted as the agonistic tetrad), have been proposed in recent studies as a putative mechanism of the agonistic effect in OX2R [23]. These four residues form two pairs, Thr111-Tyr354 and Asp115-His350, through hydrogen bonds, but their role in OX2R activation remain unclear. According to Nagahara et al., interactions of the sulfonylamide group of the agonist they developed with hydroxyl groups of Thr111 and Tyr354 and the imidazole ring of His350 may be associated with the receptor activation, although the mutual interactions of the agonistic tetrad pairs can stay preserved in the activated receptor conformation. A previous study by Heifetz et al. (2013) also pointed out the importance of Tyr317 for effective activation of OX2R by orexin peptide A [24]. If Tyr317 is mutated to Ala317, activation of OX2R by orexin peptide A is decreased by nearly 50%. In addition, it seems that blocking the contacts between the transmembrane helix 5 (TM5) and 6 (TM6) by a ligand may cause an antagonistic effect because the inward motion of the two helices, TM5 and TM6, is also related with OX2R activation. Other theories speculate that the inactivated form of OX2R is maintained by salt bridges between four residue pairs, Asp115-His350, Glu118-Arg339, Asp211-Arg328, and Glu212-His224, which stabilize the extracellular domain of OX2R [25]. Analyses of the experimental binding mode of suvorexant in OX2R (i.e., in 4S0V) prove that interactions within the agonistic tetrad (Figure S2) and pairing of the above-mentioned residues stabilizing the conformation of OX2R (Figure S3) are preserved in the inactive receptor, and the antagonist predominantly interacts with the terminal amino groups of Gln134, Gln187, and Asn324 (Figure 1). Therefore, it is probable that if the complete agonistic tetrad does not interact with the ligand, OX2R stays inactivated.

Since numerous signaling pathways for orexin receptors have been detected and carefully mapped in the CHO-K1 cell line, we chose, as the experimental system, CHO-K1 cells stably expressing OX2R. Measurement was based on the Ca^2+^ release from the intracellular storage and extracellular Ca^2+^ influx upon activation of the OX2R by a ligand. Such release can be monitored in situ via the increase of fluorescence mediated by complex formation of the intracellular Ca^2+^ with the Fluo-3 probe. 

To date, the known agonistic ligands for orexin receptors are the native or synthetic mimics of orexin A and B and several small molecules [23,26,27]. In the current study, orexin A revealed a strong concentration-dependent response for Ca^2+^ elevation (Figure S4). The EC_50_ of orexin A in OX2R was obtained as 35 ± 5 nM (n = 3), which correlates well with the literature data [3,28].

The last 15 years have witnessed the development of some orexin receptor antagonists, for example, by Actelion—structures based on glycine sulfonamide motifs [29], almorexant, or SB849868 [30]. Turku et al. indicated a dual OX1R/OX2R or OX2R–selective antagonistic effect of seven compounds with an IC_50_ range of 0.07–0.8 µM to OX2R in competition measurement with [^125^I]-orexin A [31]. In addition, suvorexant was the first orexin receptor antagonist drug recently approved for the treatment of insomnia, and, in the current study, it was used as a control [32]. Indeed, orexin A induced Ca^2+^ elevations were strongly blocked by suvorexant (IC_50_ 71 ± 13 nM, n = 3; Figure S5). Again, our data are in line with those previously reported by Winrow et al. (IC_50_ of suvorexant for human OX2R was 55 nM) [33]. In our experimental conditions, all the ligands, **L1**–**L11**, were initially tested if they showed any agonistic profile (Figure S6). However, none of the tested ligands showed an agonistic effect at 10 μM. Higher concentrations of the compounds were not tested due to their limited solubility. Compounds, **L6**, **L9**, and **L11**, showed a subtle agonistic effect during the screening and were further analyzed at higher concentrations, but neither an agonistic nor dose-response effect was eventually reproduced and confirmed (data not shown). 

We therefore turned our attention to the antagonistic effect of the ligands. Firstly, we screened the effect of all ligands at 10 μM to 100 nM of orexin A. The results showed that ligands, **L3**, **L4**, **L6**, **L7**, and **L10**, were able to significantly reduce the signal of orexin A response (data not shown). Therefore, these active compounds were investigated in serial dilution in order to obtain their IC_50_ values, which are summarized in Table 3. In this regard, **L4** can be highlighted as the most potent antagonist of orexin A with an IC_50_ of 2.2 ± 0.47 µM.

**Figure 2:**
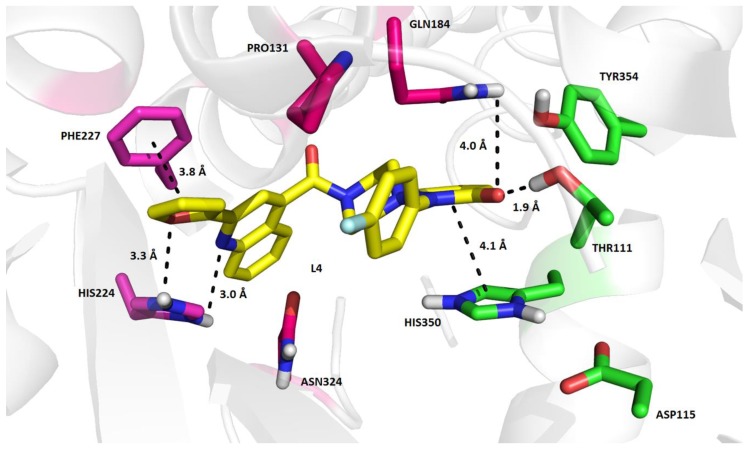
Predicted binding mode of **L4** in the OX2R model (PDB ID: 4S0V) by AutoDock Vina. The green colored residues represent the putative agonistic tetrad proposed by Nagahara et al. [23].

Focusing on the most active in vitro inhibitor found in this study, the binding mode of **L4** in OX2R elucidated by molecular docking in AutoDock Vina depends on several interactions, which involve both types of residues putatively considered responsible for activation as well as inhibition of the receptor. The furan heterocycle of **L4** is stabilized by a weak hydrogen bond with His224 (3.3 Å) and simultaneously by a weak distorted π-stacking with Phe227 (3.8 Å). His224 also contributes to the binding affinity of **L4** by a weak hydrogen bond with the nitrogen atom of the quinoline moiety (3.0 Å). A significant stabilization element of the **L4** binding mode in OX2R is formed by a hydrogen bond of the pyridazin-3(2*H*)-one moiety with Gln184 and Thr111 (4.0 Å, 1.9 Å). In addition, this structural part of **L4** interacts also by a shifted π-stacking with His350. Although **L4** exhibited a relatively strong *in silico* binding affinity for OX2R (e.g., −15.0 kcal/mol), its interactions with only two residues of the agonistic tetrad (e.g., Thr111 and His350) are likely insufficient to trigger the agonistic cascade reaction. Inactivation of OX2R may be partially supported by the fact that neither suvorexant nor **L4** interact with Tyr317. Comparing the inactivated forms of OX2R, mutual conformation of the agonistic tetrad residues is nearly the same in OX2R inhibited by suvorexant and **L4**. Similarly, no large conformational changes of residue pairs, Asp115-His350, Glu118-Arg339, Asp211-Arg328, and Glu212-His224, between OX2R inhibited by suvorexant and docked with **L4** can be observed. On the other hand, the binding mode of **L4** and the value of its *in silico* binding affinity for OX2R sheds some light on the fact that this ligand prevents the in vitro interaction of orexin peptide A with OX2R quite significantly, even though it is not interacting with Asn324 and Gln187, like suvorexant (Figure 2). 

Unfortunately, the binding affinities of **L3**, **L4**, **L6**, **L7**, and **L10** for OX2R predicted by AutoDock Vina correlate only weakly with their logarithmized in vitro IC_50_ values (R^2^ = 0.24). Although the above-mentioned computational chemistry simulations enabled the selection of promising lead candidates for OX2R modulation, the reported results can only serve as simplified qualitative representations of the putative mechanisms of action. More advanced calculations, such as molecular dynamics with improved binding energy estimation (e.g., free energy perturbation) and implementation of the solvent effects, would be necessary to refine the quantitative agreement between the *in silico* and in vitro results. However, the present molecular docking studies can be taken as a necessary starting point for more advanced calculations.

Although we did not study the ligand-OX2R interactions with quantum mechanics methods, which are the most accurate, we did construct a molecular dynamics-refined homology model of OX2R based on thhe PDB ID 5WQC structure. Docking of suvorexant and **L1**–**L11** into the model in AutoDock Vina and subsequent analysis of the obtained poses with LigPlot suggested the possible occurrence of several non-obvious interactions, which overlapped with those found with the FMO (Fragment Molecular Orbital) method by Heifetz et al. [24]. The ones corresponding to interacting residues found by Heifetz et al. were H-bonds with Gln134, Glu212, Ile320, Asn324, and His350, and hydrophobic interactions with Pro131, Gln134, Gln187, Glu212, Phe227, Ile 320, Asn234, His350, and Tyr354.

One of the major demands of novel OX2R modulators is their ability to permeate the blood-brain barrier (BBB). Accordingly, the capability of **L1**–**L11** was predicted using the parallel artificial membrane permeability assay (PAMPA). It is a high-throughput screening tool applied to predict the passive transport of potential drugs across the BBB [34]. All the data obtained are listed in Table 4. Selected standard drugs with known BBB permeability were used as controls. The studied ligands with values of *Pe* over 4.0 × 10^−6^ cm s^−1^ (except for **L7** and **L8**) are considered as substances with high probability to cross the BBB. **L7** and **L8** fall in the interval of *Pe* 2.0–4.0 × 10^−6^ cm s^−1^, with uncertain BBB permeation via passive diffusion [34].

Another important attribute of new lead candidates is their toxicity profile. Hence, the studied ligands, **L1**–**L11**, were also evaluated for their cytotoxicity by standard in vitro cell viability MTT (3-(4,5-dimethylthiazol-2-yl)-2,5-diphenyltetrazolium bromide) colorimetric assay. In line with the diverse chemical structures of the studied compounds, widely-varying values of IC_50_ were observed, as summarized in Table 4. The lowest cytotoxicity was exhibited by **L8** (IC_50_ over ~700 µM). Observed antagonistic effects of compounds was concentration-dependent. These were effective at lower concentrations than that identified for cellular toxicity. Considering this fact, we expected that colloidal aggregation, which can lead to residual activity against G-protein coupled receptors [35], is improbable. These experimental observations disproving the compounds’ aggregation at the applied concentrations are in a good agreement with *in silico* predicted potency of aggregation (http://advisor.bkslab.org). Using ligand-based similarity tests, only compounds, **L3**, **L4**, and **L5**, exhibited approximately 70% of maximal structural similarity with the known aggregators (Table S1).

## 3. Materials and Methods

### 3.1. Structure-Based Virtual Screening

Discovery of unknown OX2R modulators was accomplished by structure-based virtual screening (SBVS) using iDock, AutoDock Vina 1.1.2 freeware (open-source program, New York, NY, USA) and a peta-flops-scale supercomputer [36]. Our preliminary computational research concerning SBVS for OX2R modulators has already been published, and this study utilizes its predictions [37]. In the SBVS, the initial pool of virtual ligands contained 1,000,448 randomly selected compounds belonging to the All Clean subset of purchasable drug-like substances stored in the ZINC database (zinc.docking.org). These compounds were transformed into pdbqt format by AutoDock Tools 1.5.4 freeware and docked into the OX2R model (PDB ID: 4S0V) by iDock freeware (http://istar.cse.cuhk.edu.hk/idock/), assigning torsional flexibility only on the ligands. The receptor OX2R was protonated, split into rigid and flexible parts, and prepared for docking also in AutoDock Tools 1.5.4. as appropriate. In general, AutoDock tools detects torsional bonds, merges non-polar hydrogens with carbons, and assigns atom types and Gasteiger charges. The gridbox design was based on the topology of suvorexant in the 4S0V model, which enabled localization of the gridbox center in point x = 52 Å, y = 8 Å, z = 53 Å. The resulting binding energy estimates were used for selection of the 1000 top-scoring candidates, which were subsequently re-docked by AutoDock Vina 1.1.2 in the same OX2R model, involving torsional flexibility not only for the ligands, but also for 38 selected residues in the active site of the receptor (i.e., selection of 38 residues closest to suvorexant in 4S0V, which were involved in the gribdox of a size of 17 × 14 × 16 Å). The 11 top-scoring ligands with the lowest binding energy estimates provided by AutoDock Vina were purchased and biochemically tested for their in vitro activity in OX2R. The binding modes obtained by molecular docking were illustrated in PyMOL 1.6.

### 3.2. Chemicals

All chemicals and reagents were purchased in reagent grade quality and used without further purification. Bovine Serum Albumin (BSA), calcium chloride, d-(+)-Glucose, Dulbecco’s Phosphate Buffered Saline (PBS), fetal bovine serum, gelatin, magnesium sulfate heptahydrate, 3-(4,5-dimethylthiazol-2-yl)-2,5-diphenyltetrazolium bromide (MTT), Pluronic^®^ F-127, potassium chloride, Probenecid, sodium chloride, tris(hydroxymethyl)aminomethane (Tris), and trypsin-EDTA solution were obtained from Sigma Aldrich (Prague, Czech Republic). Antibiotic G418, antimycotin solution, penicillin-streptomycin, and zeocin were also purchased from Sigma Aldrich (Prague, Czech Republic). Fluo-3 AM was purchased from Thermo Fisher Scientific (Pardubice, Czech Republic), orexin A human, rat, mouse from Bachem (Bubendorf, Switzerland), and suvorexant from Cayman Chemical (Neratovice, Czech Republic). 

The hit structures resulting from SBVS were purchased from MolPort (Riga, Latvia, **L1**–**L5**, **L8**), Vitas-M Laboratory (Apeldoorn, the Netherlands, **L6** and **L7**), and Mcule (Budapest, Hungary, **L9**–**L11**). All the structures **L1**–**L11** were analyzed by a Dionex UltiMate 3000 analytical UPLC system coupled with a Q Exactive Plus hybrid quadrupole-orbitrap spectrometer (both produced by Thermo Fisher Scientific, Bremen, Germany) to confirm their identity and uncalibrated purity at the wavelength of 254 nm. According to the analyses, all the compounds exhibited [M + H]^+^ differing at maximum by ±0.001 from the theoretical exact mass and proved uncalibrated purity in the range from 89.26 to 99.87%. (Table S2).

### 3.3. Cell Cultures

Adherent epithelial CHO-K1 cell line (Chinese hamster ovary, ATCC, #CCL-61, Manassas, VA, USA) was grown in Ham’s F12 Nutrient Mixture supplemented with 10% heat-inactivated FBS and antibiotics 1% penicillin (10,000 U/mL)/streptomycin (10,000 µg/mL) at 37 °C, 95% humidity and under 5% CO_2_. 

CHO-K1 cell line stably expressing human OX2R receptor (Perkin Elmer, Norwalk, CT, USA) was grown in Ham’s F12 Nutrient Mixture supplemented with 10% heat-inactivated FBS and antibiotics G418 (0.4 mg/mL) and zeocin (0.25 mg/mL) at 37 °C, 95% humidity and under 5% CO_2_. Glutamax and antibiotic solution (1% penicillin/streptomycin/amphotericin) were added to the culture medium for the experiment. 

### 3.4. OX2R Calcium Assay

#### 3.4.1. Assay Buffer Preparation

Fresh assay buffer used for the experiment contained 5 mM glucose, 140 mM NaCl, 15 mM Tris-HCl, 3.5 mM KCl, 1.25 mM CaCl_2_, 1.2 mM PB buffer (NaH_2_PO_4_ × H_2_O and Na_2_HPO_4_ × 2H_2_O titrated to pH 7.4), 1 mM Mg_2_SO_4_, 0.1% BSA, and 0.05% gelatin.

#### 3.4.2. Calcium Assay Procedure

CHO-K1 cells stably expressing human OX2R were seeded in a 96-well black, clear-bottom sterile plate in culture medium without antibiotics (50,000 cells per well), and left overnight (37 °C, 5% CO_2_). 18 h later, 50 µL of cell culture medium was removed from each well and mixed with loading solution composed of Fluo-3 AM (Ca^2+^ sensitive fluorescent dye dissolved in DMSO, 50 µg/50 µL), 10% F-127 (1:1), and probenecid (1.5%). The cells were then loaded with Fluo-3 by adding back 50µl of the loading solution to each well and incubating for 45 min at 37 °C in the cell culture incubator. Afterwards, the medium with loading buffer from each well was drained and the wells were washed with washing buffer composed of assay buffer and 1.5% probenecid. The cells in the plates were then again incubated for 30 min at room temperature in the dark. The washing buffer was then drained and 180 µL assay buffer was added manually to each well. Next, the plate was placed in a multi-plate reader Synergy HT (Biotek, Winooski, VT, USA) for 5 min. In the OX2R agonist assay, the response to orexin A or hit compounds, **L1**–**L11**, was measured in the plate-mode, i.e., the baseline was recorded for each well, the orexin A or tested compound was then manually added in appropriate concentration (20 µL) to the well, and after 120 s incubation, the Ca^2+^ response was measured as fluorescence changes (λ_ex_ = 485 nm, λ_em_ = 528 nm) at 30 °C. Sustained slow effects seen from orexin A were searched in the agonistic mode. In the OX2R antagonist assay, the studied hit compounds, **L1**–**L11**, that dissolved in the assay buffer at the appropriate concentration (180 µL) were added first to the wells, and after 5 min, incubation orexin A (20 µL) was added manually. Suvorexant was used as a reference antagonist. The obtained data were transferred to Microsoft Excel and GraphPad Prism 7.03 (GraphPad Software Inc., San Diego, CA, USA) for visualization and analysis.

All results in the OX2R calcium assay are presented as mean ± SEM (the standard error of the mean) of at least three independent experiments. IC_50_ and EC_50_ values were determined using non-linear regression (log [inhibitor] vs*.* response, four parameters, symmetric). Ca^2+^ release was calculated as the ratio of fluorescence (*F*):

(1) $$\;F=\frac{F_{peak}-F_{basal}}{F_{basal}}\;$$

after subtraction of assay buffer fluorescence, where *F_basal_* and *F_peak_* are the fluorescence intensities, respectively, before and after addition of agonistic ligand, and were measured at five time points every 26 s.

### 3.5. PAMPA Assay

The parallel artificial membrane permeability assay (PAMPA) was used as the non-cell-based in vitro assay to predict BBB penetration carried out in a coated 96-well membrane filter [34]. The filter membrane of the donor plate was coated with PBL (Polar Brain Lipid, Avanti, Alabaster, AL, USA) in dodecane (4 µL of 20 mg/mL PBL in dodecane) and the acceptor well was filled with 300 µL of PBS buffer (pH 7.4; *V_A_*). Tested compounds were dissolved first in DMSO and then diluted with PBS (pH 7.4) to achieve the final concentration of 30 µM in the donor well. The concentration of DMSO did not exceed 0.5% (*v*/*v*) in the donor solution. 300 µL of the donor solution (*V_D_*) was added to the donor wells and the donor filter plate was carefully put on the acceptor plate so that coated membrane was “in touch” with both the donor solution and acceptor buffer. The test compound diffused from the donor well through the polar brain lipid membrane (Area = 0.28 cm^2^) to the acceptor well. The concentration of the tested compound in both the donor and acceptor wells was assessed after 3, 4, 5, and 6 hours of incubation, respectively, in quadruplicate using a UV plate reader Synergy HT (Biotek, USA) at the maximum absorption wavelength of each compound. Also prepared were solutions at the theoretical equilibrium of the given compound (i.e., the theoretical concentration if the donor and acceptor compartment were simply combined). Concentrations of the compounds in the donor and acceptor well and the equilibrium concentration were calculated from the standard curve and expressed as the permeability (*Pe*) according to the equation [34]:

(2) $$Pe=C\times -ln\left(1-\frac{{\left[drug\right]}_{acceptor}}{{\left[drug\right]}_{equilibrium}}\right),$$

where

(3) $$C=\left(\frac{V_{D}\times V_{A}}{\left(V_{D}+V_{A}\right)\times Area\times Time}\right).$$

### 3.6. MTT Assay

The cytotoxic effect of the studied compounds was assessed using standard MTT (3-[4–dimethylthiazol-2-yl]-2,5-diphenyltetrazolium bromide) assay on the CHO-K1 cell line. The tested compounds (100–700 µM) were dissolved in DMSO and subsequently in the growth medium, such that the final DMSO concentration did not exceed 0.5% (*v*/*v*). Cells were seeded in 96-well plates and exposed to the tested compounds in the medium (100 µL) for 24 h at 37 °C, 95% humidity, and in an atmosphere of 5% CO_2_. Subsequently, this medium was replaced by the medium containing MTT (10 µM) and incubated for another 3–4 h. After that, the medium with MTT was removed and formazan crystals were dissolved by the addition of 100 µL DMSO. Viability of the cells was estimated spectrophotometrically by the amount of produced formazan. The absorbance was measured at 570 nm, with 650 nm as a reference wavelength on a Synergy HT reader (Biotek, USA). Results were expressed as IC_50_ from the control-subtracted triplicates (in comparison with untreated control) using non-linear regression (four parameters) in GraphPad Prism 7.03 (GraphPad Software Inc., CA, USA). 

## 4. Conclusions

In summary, we have applied *in silico* screening to obtain novel lead candidates targeting OX2R. Initially, our attempts sought to identify a novel receptor agonist. Instead, we have discovered a novel OX2R antagonist, highlighting **L4**, which still offers some promise mainly for the treatment of insomnia, and supported by low cytotoxicity and predicted CNS availability. The study also disclosed the central availability of almost all the compounds except for two of them (**L7** and **L8**), with uncertain permeation through the BBB. Interestingly, the compounds displayed a low cytotoxicity profile, with one to two-digit micromolar IC_50_ values in the CHO-K1 cell line. We believe that the current study represents a promising starting point to follow, and it can also help in designing novel compounds targeting OX2R in the treatment of insomnia.

## Figures and Tables

**Table 1:** Chemical characteristics of the studied compounds, **L1**–**L11**.

Codename	IUPAC Name	Chemical Structure	Mw [g mol^−1^]	log*P* ^1^
**L1**	1-(1-{5*H*,6*H*-benzo[*h*]quinazolin-2-yl}-5-cyclopropyl-1*H*-pyrazole-4-carbonyl)-4-benzylpiperazine	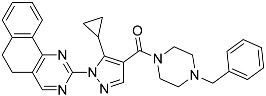	490.60	4.90
**L2**	4-{2-[4-(naphthalene-2-sulfonyl)piperazin-1-yl]acetyl}-1,2,3,4-tetrahydroquinoxalin-2-one	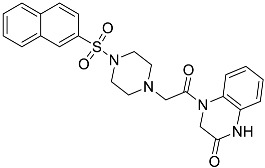	464.54	1.58
**L3**	4-[4-(2,3-dihydro-1,4-benzodioxine-2-carbonyl)piperazine-1-carbonyl]-2-(furan-2-yl)quinoline	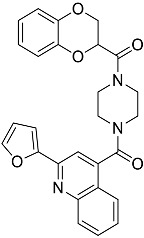	469.49	3.00
**L4**	2-(4-fluorophenyl)-6-{4-[2-(furan-2-yl)quinoline-4-carbonyl]piperazin-1-yl}-2,3-dihydropyridazin-3-one	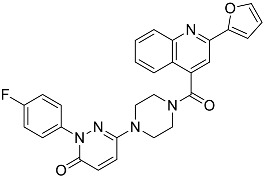	495.50	3.60
**L5**	(12*E*)-22-hydroxy-20-(2-hydroxyquinolin-3-yl)-4-methyl-3,17-dioxatricyclo[12.8.0.0^16,21^]docosa-1(14),12,15,21-tetraene-2,8,18-trione	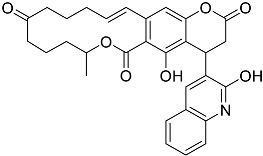	515.55	6.46
**L6**	3,5-dimethyl-13-(4-oxo-4*H*-chromen-3-yl)-8-phenyl-12-oxa-3,5,9-triazatricyclo[7.4.0.0^2,7^]trideca-1,7-diene-4,6-dione	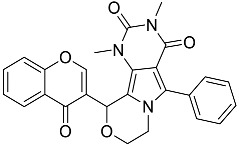	455.46	2.69
**L7**	(6*E*)-5-imino-6-({1-[(2-methylphenyl)methyl]-1*H*-indol-3-yl}methylidene)-2-[2-oxo-2-(pyrrolidin-1-yl)ethyl]-5*H*,6*H*,7*H*-[1,3,4]thiadiazolo[3,2-a]pyrimidin-7-one	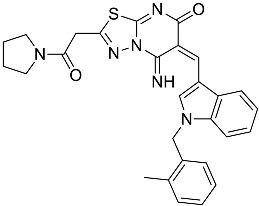	510.61	4.47
**L8**	*N*-[(1R,9*S*)-6-oxo-11-(pyridine-2-carbonyl)-7,11-diazatricyclo[7.3.1.0^2,7^]trideca-2,4-dien-5-yl]benzamide	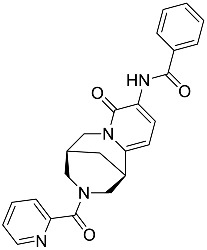	414.46	1.14
**L9**	3-{2-[2-(3,4-dihydro-2*H*-1,5-benzodioxepin-7-yl)pyrrolidin-1-yl]-2-oxoethyl}-5-(4-fluorophenyl)-2,3-dihydro-1,3,4-oxadiazol-2-one	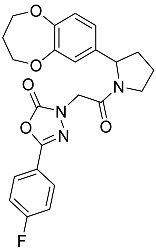	439.44	2.91
**L10**	*N*-[3-(2,3-dihydro-1-benzofuran-5-yl)phenyl]-2-oxo-1,2,3,4-tetrahydroquinoline-4-carboxamide	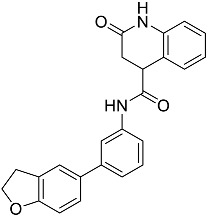	384.435	3.70
**L11**	*N*-[2-(2,5-difluorophenyl)-1*H*-1,3-benzodiazol-5-yl]-1-ethyl-1*H*-1,2,3-benzotriazole-5-carboxamide	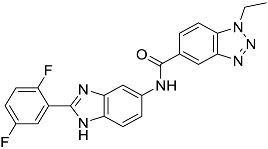	418.40	4.47
Suvorexant	5-chloro-2-{3-methyl-4-[5-methyl-2-(2*H*-1,2,3-triazol-2-yl)benzoyl]piperazin-1-yl}-1,3-benzoxazole	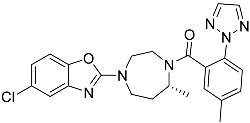	450.92	3.99

^1^ calculated with MarvinSketch 17.17.0.

**Table 2:** Binding energy estimates predicted by iDock and AutoDock Vina 1.1.2 software. The ligand molecules were docked in a model of orexin receptor 2(OX2R) available under PDB ID: 4S0V in the online rcsb.org database.

Ligand	Binding Energy Estimate [kcal/mol]	
	iDock	AutoDock Vina
**L1**	−11.8	−13.7
**L2**	−11.5	−14.8
**L3**	−12.0	−13.6
**L4**	−11.9	−15.0
**L5**	−12.4	−15.4
**L6**	−12.1	−13.5
**L7**	−11.8	−14.6
**L8**	−11.0	−12.3
**L9**	−11.2	−13.2
**L10**	−12.2	−13.7
**L11**	−12.1	−13.8
Suvorexant	−11.1	−12.5

**Table 3:** Antagonistic effect of ligands, **L3**, **L4**, **L6**, **L7**, and **L10**, and suvorexant in orexin A-OX2R interaction. IC_50_ values were calculated as mean ± SEM (n = 3) ^1^.

Ligand	IC_50_ µM ± SEM
**L3**	8.9 ± 0.43
**L4**	2.2 ± 0.47
**L6**	12.2 ± 3.1
**L7**	25 ± 2.6
**L10**	27.8 ± 0.5
Suvorexant	0.071 ± 0.013

^1^ 0.1 μM orexin A was used as agonist of OX2R for revelation of the antagonistic effect of the studied ligands.

**Table 4:** Prediction of the blood-brain barrier penetration and cytotoxicity data of studied compounds, **L1**–**L11**.

Ligand	*Pe* ± SEM (×10^−6^ cm s^−1^) ^1^	CNS Predicted Availability ^2^	Cytotoxicity CHO-K1 IC_50_ µM ± SEM
**L1**	10.2 ± 0.9	CNS +	78.8 ± 6.88
**L2**	31.6 ± 3.4	CNS +	165 ± 5.36
**L3**	17.9 ± 2.2	CNS +	279 ± 36.6
**L4**	18.3 ± 0.1	CNS +	99.8 ± 10.7
**L5**	12.0 ± 2.5	CNS +	59.0 ± 6.41
**L6**	17.6 ± 6.4	CNS +	>100
**L7**	3.37 ± 2.1	CNS +/−	61.8 ± 3.74
**L8**	4.0 ± 0.3	CNS +/−	~700
**L9**	32.3 ± 1.5	CNS +	143.6 ± 11.1
**L10**	18.9 ± 2.4	CNS +	53.9 ± 5.03
**L11**	ND	ND	28.3 ± 4.07
Tacrine	6.0 ± 0.6	CNS +	ND
Donepezil	21.9 ± 2.1	CNS +	ND
Rivastigmine	20.0 ± 2.1	CNS +	ND
Ibuprofen	18.0 ± 4.3	CNS +	ND
Chlorothiazide	1.1 ± 0.5	CNS −	ND
Furosemide	0.2 ± 0.07	CNS −	ND
Ranitidine	0.04 ± 0.02	CNS −	ND
Sulfasalazine	0.09 ± 0.05	CNS −	ND

^1^ The results are the mean of at least three independent measurements ± SEM; ND = not determined due to low solubility; ^2^ CNS + (high BBB permeation predicted): *Pe* (×10^−6^ cm s^−1^) > 4.0; CNS − (low BBB permeation predicted): *Pe* (×10^−6^ cm s^−1^) < 2.0; CNS +/− (BBB permeation uncertain): *Pe* (×10^−6^ cm s^−1^) from 4.0 to 2.0 [34].

## Supplementary Materials

The following are available online, Figure S1: Superposition of the suvorexant binding mode in OX2R determined by X-ray (colored in magenta, PDB ID: 4S0V) and by molecular docking in AutoDock Vina (colored in green), Figure S2: Interactions among the residues of agonistic tetrad (colored in green) in OX2R inhibited by suvorexant (i.e., PDB ID: 4S0V), Figure S3: Interactions of other residues in OX2R inhibited by suvorexant (PDB ID: 4S0V), which are considered to stabilize inactivated conformation of OX2R, Figure S4: Typical dose-response effect by orexin A (0–500 nM) stimulation, Figure S5: Typical dose-response effect of suvorexant after orexin A (100 nM) stimulation, Figure S6: The screening of agonistic activity of ligands **L1**–**L11** (10 µM) on OX2R, Table S1: *In silico* analysis of aggregation potency by Tanimoto similarity (TS) with known aggregators (http://advisor.docking.org/), Table S2: Evaluation of identity and uncalibrated purity of **L1**–**L11** with LC-UV-HRMS.
