# Supplementary material for: Investigation of New Orexin 2 Receptor Modulators Using In Silico and In Vitro Methods
Source: Molecules. 2018 Nov 9;23(11):2926. doi: 10.3390/molecules23112926 (PMC6278354; doi:10.3390/molecules23112926)
Supplement: Supplementary file 1 [file molecules-23-02926-s001.pdf]

molecules

MDPI

Supplementary information

Investigation of new orexin 2 receptor modulators using *in silico* and *in vitro* methods

Jana Janockova [1, 3, #], Rafael Dolezal [1, 3, 5, #], Eugenie Nepovimova [3], Tereza Kobrlova [1, 2], Marketa Benkova [1], Kamil Kuca [1, 3], Jan Konecny [1, 2], Eva Mezeiova [1], Michaela Melikova [3], Vendula Hepnarova [1, 2], Avi Ring [4], Ondrej Soukup [1, *] and Jan Korabecny [1, *]

[1] Biomedical Research Centre, University Hospital Hradec Kralove, Sokolska 581, 500 05 Hradec Kralove, Czech Republic; jana.janockova@fnhk.cz (J.J.), Rafael.dolezal@fnhk.cz (R.D.) ,tereza.kobrlova@fnhk.cz (T.K.), marketa.benkova@fnhk.cz (M.B.), kamil.kuca@fnhk.cz (K.K.), jan.konecny@fnhk.cz (J.Kon.), eva.mezeiova@fnhk.cz (E.M.),ondrej.soukup@fnhk.cz (O.S), jan.korabecny@fnhk.cz (J.Kor.)

[2] Department of Toxicology and Military Pharmacy, Faculty of Military Health Sciences, University of Defence, Trebesska 1575, 500 05 Hradec Kralove, Czech Republic; vendula.hepnarova@unob.cz (V.H.)

[3] Department of Chemistry, Faculty of Science, University of Hradec Kralove, Rokitanskeho 62, 500 03 Hradec Kralove, Czech Republic; eugenie.nepovimova@uhk.cz ,(E.N), michaela.melikova@upol.cz (M.M)

[4] Norwegian Defence Research Establishment, Gunnar Randersvei 42, 2007 Kjeller, Norway; avi.ring@ffi.no

[5] Center for Basic and Applied Research, University of Hradec Kralove, Rokitanskeho 62, 500 03 Hradec Kralove, Czech Republic;

# J.J. and R.D. contributed equally

* Correspondence: jan.korabecny@fnhk.cz, Tel.: +420-973-255-167; ondrej.soukup@fnhk.cz, Tel.: +420-495-833-447

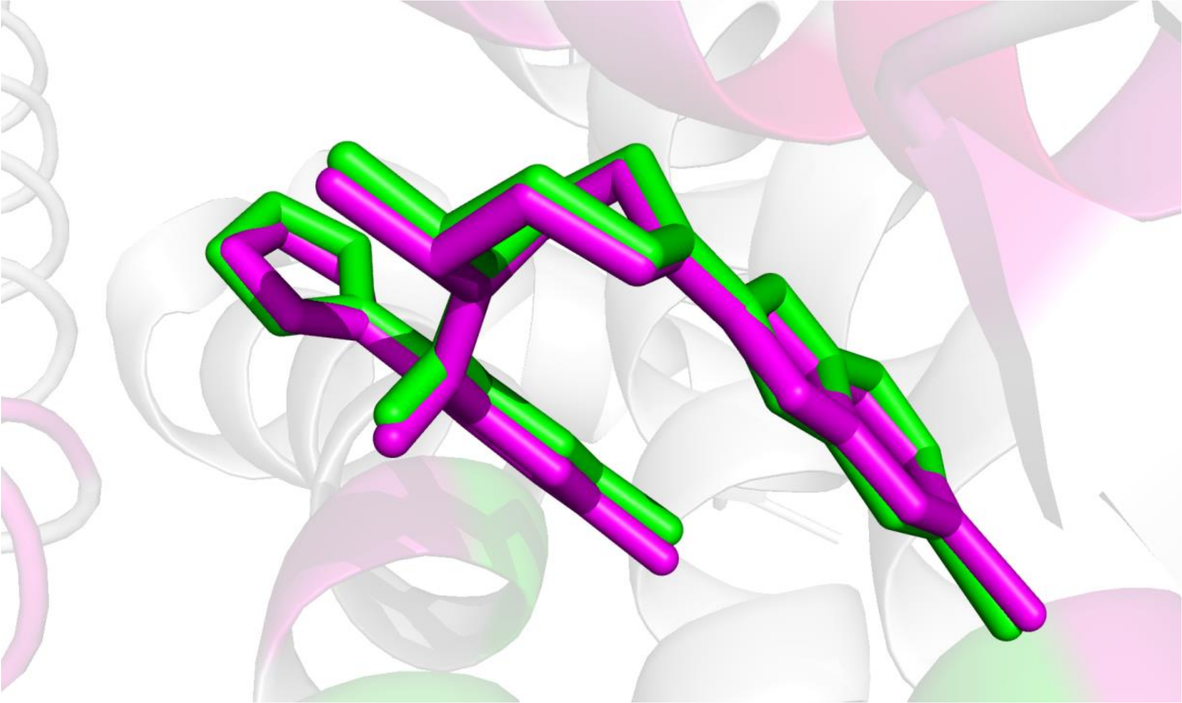

Figure S1. Superposition of the suvorexant binding mode in OX2R determined by X-ray (colored in magenta, PDB ID: 4S0V) and by molecular docking in AutoDock Vina (colored in green). The resulting RMSD of the two poses is 0.185 Å.

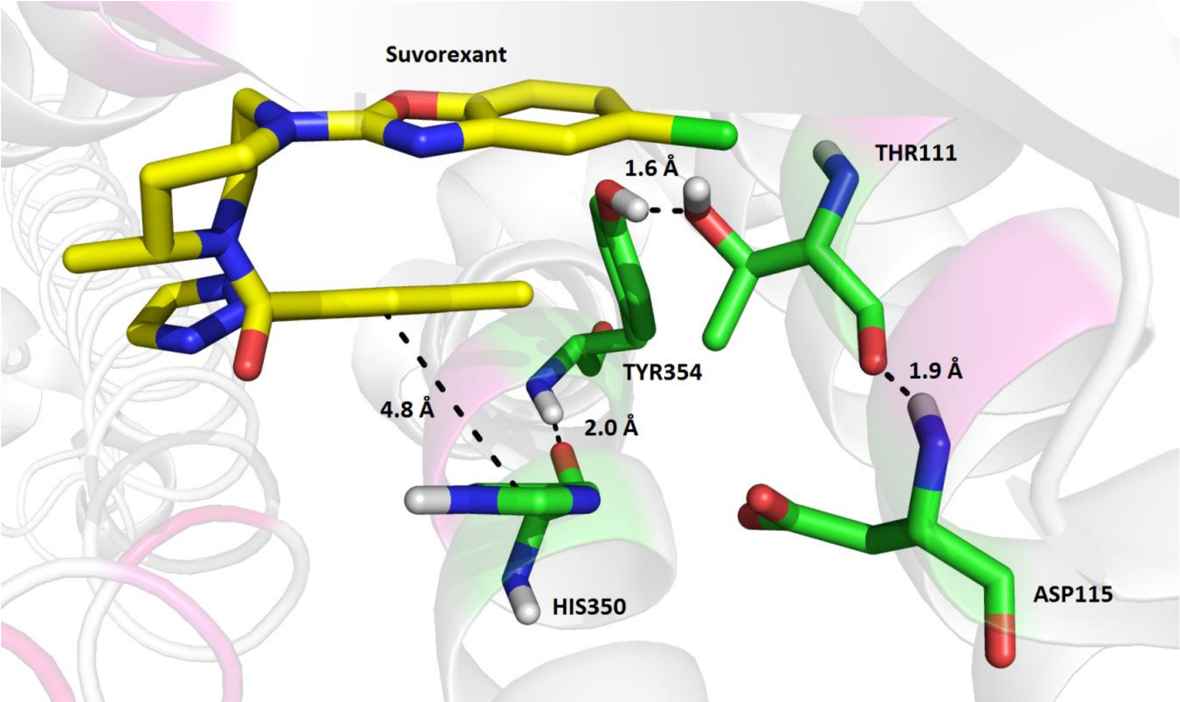

Figure S2. Interactions among the residues of agonistic tetrad (colored in green) in OX2R inhibited by suvorexant (i.e. PDB ID: 4S0V).

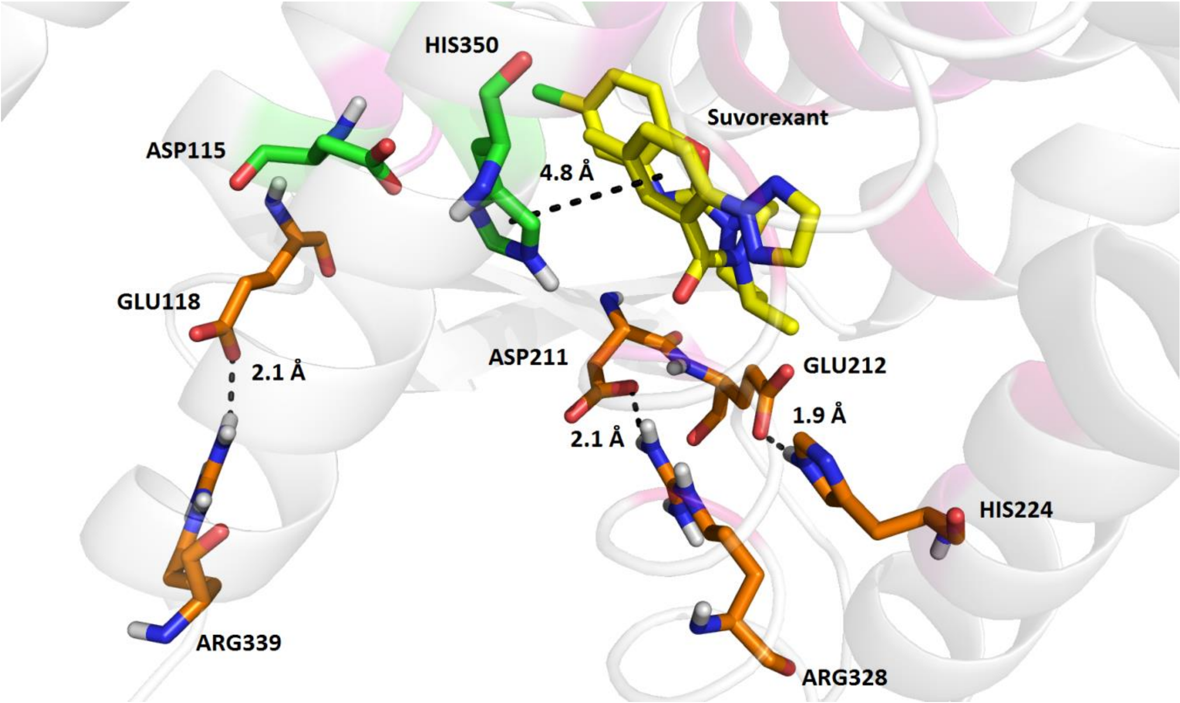

Figure S3. Interactions of other residues in OX2R inhibited by suvorexant (PDB ID: 4S0V), which are considered to stabilize the inactivated conformation of OX2R.

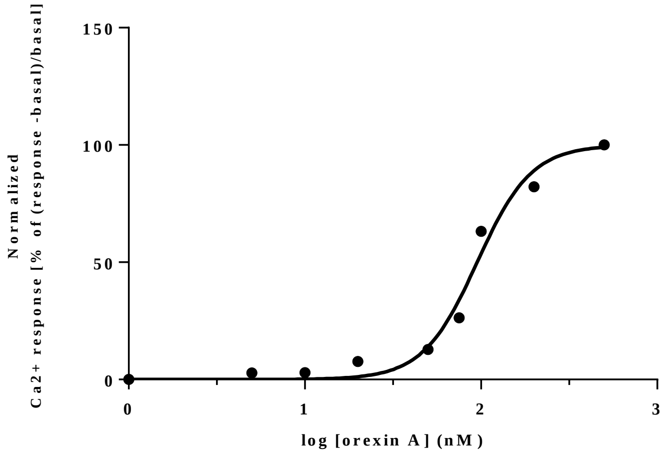

Figure S4. Typical dose-response effect by orexin A (0 - 500nM) stimulation. Responses are presented as fluorescence increase above the baseline (response-basal)/basal and are normalized to the baseline (0%) and the maximum concentration of orexin A (100%) at F_{485nm}/F_{528nm}. Values are the means of triplicate.

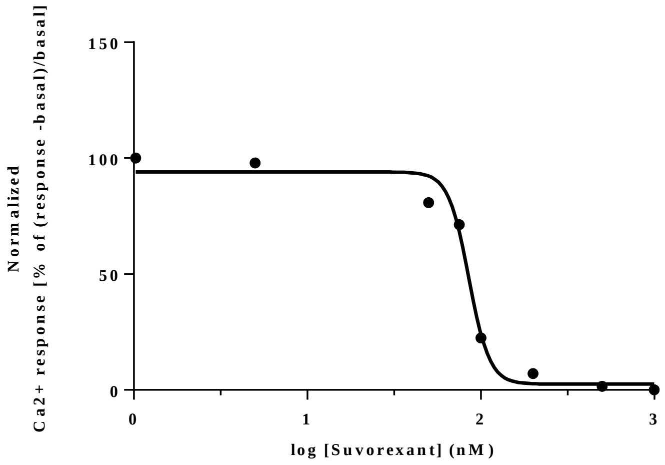

Figure S5. Typical dose-response effect of suvorexant after orexin A (100 nM) stimulation. Responses are presented as (response-basal)/basal and are normalized to the baseline (100%) and the maximum concentration of suvorexant (0%) at F_{485nm}/F_{528nm}. Values are the means triplicate.

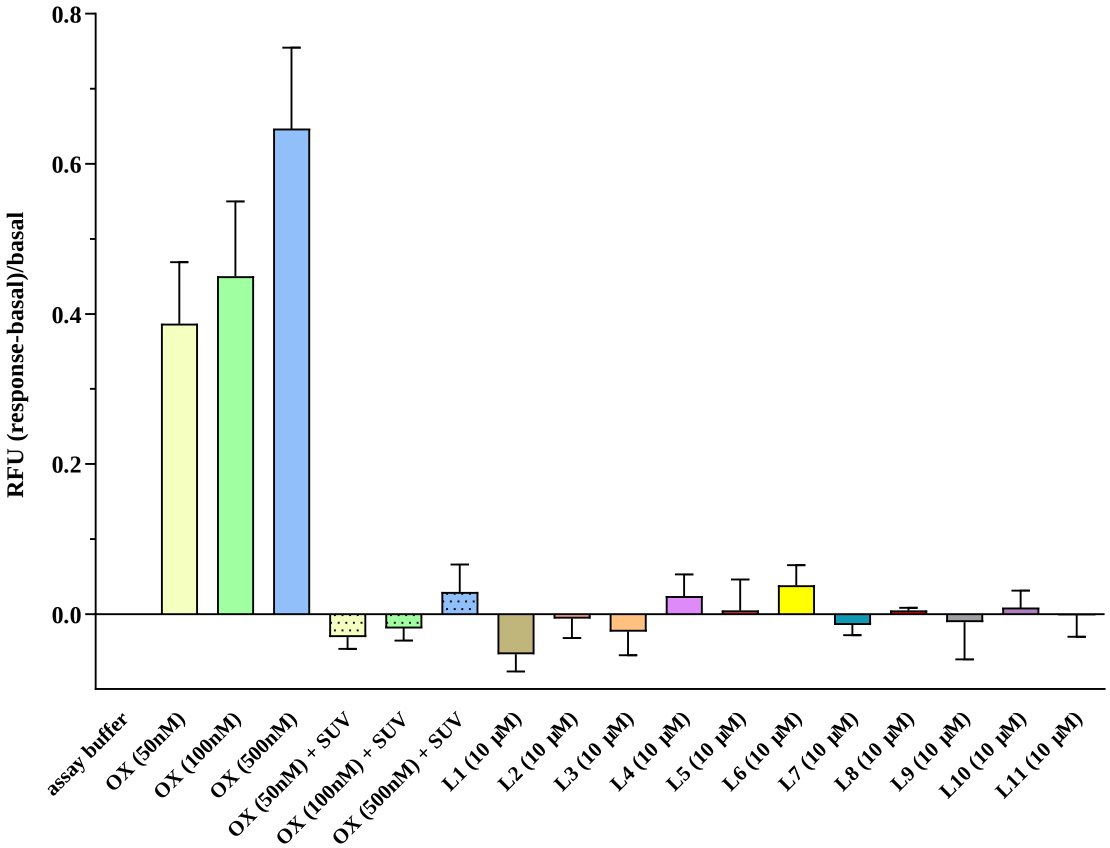

Figure S6. The screening of agonistic activity of ligands **L1-L11** (10 µM) on OX2R. OX = orexin, SUV = suvorexant.

Table S1. *In silico* analysis of aggregation potency by Tanimoto similarity (TS) with know aggregators (http://advisor.docking.org/).

Ligand	TS with known aggregators
L1	- [1]
L2	71 %
L3	70
L4	- [1]
L5	- [1]
L6	71 %
L7	- [1]
L8	- [1]
L9	- [1]
L10	- [1]
L11	- [1]
Suvorexant	- [1]

[1] The test revealed no significant TS of the structure with known aggregators.

Table S2. Evaluation of identity and uncalibrated purity of **L1** – **L11** with LC-UV-HRMS.

Ligand	Predicted [M+H]$^+$	Measured [M+H]$^+$	Uncalibrated purity at 254 nm [%]
**L1**	491.25539	491.25531	97.29
**L2**	465.15910	465.15897	96.06
**L3**	470.17105	470.17126	94.35
**L4**	496.17794	496.17755	92.31
**L5**	516.20168	516.20197	89.26
**L6**	456.15540	456.15527	99.04
**L7**	511.19107	511.19110	99.31
**L8**	415.17647	415.17645	98.29
**L9**	440.16163	440.16141	99.87
**L10**	385.15467	385.15463	94.59
**L11**	419.14264	419.14236	94.05
